# The impact of sports event brand experience on spectator loyalty: mediating roles of attitude and subjective norms

**DOI:** 10.1038/s41598-025-15491-x

**Published:** 2025-08-21

**Authors:** Baoxia Chen, Zhenguo Shi

**Affiliations:** College of Physical Education, Shandong University, jinan, China

**Keywords:** Sports event brand experience, Spectator loyalty, Subjective norms, Partial least squares method, Psychology, Human behaviour

## Abstract

Sports events play a vital role in driving mass fitness, competitive sports, and the sports industry. Grounded in the Theory of Reasoned Action, this study surveyed 1,009 Chinese sports event spectators to examine how brand experience influences spectator loyalty, focusing on the mediating roles of attitude and subjective norms and the moderating effect of gender. Structural equation modeling revealed that brand experience has a significant direct effect on loyalty. Subjective norms partially mediate this relationship, while attitude alone does not show significant mediation. The chain mediation through both attitude and subjective norms is partially significant. Gender moderates the pathway from brand experience to subjective norms, with women being more influenced than men. The findings suggest that in collective consumption contexts, subjective norms play a more critical role than attitude in mediating loyalty. This study extends the application of the Theory of Reasoned Action in sports tourism and provides insights for differentiated event brand management and destination promotion strategies.

## Introduction

In recent years, China’s sports market has entered a period of steady development, driven by the major international events such as the 24th Beijing Winter Olympics, the 19th Hangzhou Asian Games, and the 31 st Chengdu Universiade. These events have not only served as a strategic response to China’s “dual circulation” policy but have also significantly advanced the development of mass fitness, elite sports, and the broader sports industry. At the policy level, the “14th Five-Year Sports Development Plan” and the 2024 National Sports Industry Work Conference have explicitly emphasized enhancing the brand value of sports events as a strategic objective to achieve high-quality development within the sports sector. These macro-level policies position event brands as key platforms through which national strategies are translated into consumer engagement. In parallel with policy developments, the global rise of the experience economy^1^ has transformed spectator expectations from functional utility toward experiential value. Within the sports industry specifically, this transformation manifests as increasing demand for immersive experiences that combine sensory stimulation, emotional engagement, and social connection^2^. The convergence of policy initiatives and consumer preferences has positioned sports event brand experiences as a bridge between national objectives and spectator satisfaction, reflecting a fundamental shift from valuing competitive outcomes to appreciating experiential platforms. This evolution marks a shift functional attributes toward co-created experiences that engage spectators across multiple touchpoints, establishing brand experience as a critical determinant of spectator loyalty. Research on sports event brand experiences has undergone significant development over two decades, beginning with examinations of team identification and brand associations^3,4^advancing to investigations of service quality and event atmosphere^5^and extending to explorations of psychological mechanisms such as satisfaction^6^ and emotional attachment^7^. Research methodologies have similarly evolved from basic correlation analyses toward sophisticated structural modeling approaches^8^. Despite this growing interest, existing research has primarily focused on the direct effects of brand experience on loyalty^9^with limited attention to the underlying mechanisms that explain this relationship or the conditional factors that might strengthen or weaken it. A more comprehensive understanding of these mediating processes and boundary conditions is essential for both theoretical development and effective brand management in the increasingly competitive sports event marketplace. While contemporary approaches recognize the multidimensional nature of sports consumption, the underlying mechanisms and key moderating factors remain underexplored.

The Theory of Reasoned Action (TRA) provides a theoretical lens for understanding how individual behavior is driven by two primary factors: attitudes and subjective norms^10^. In the highly socialized consumption context of sports events, spectator loyalty is shaped not only by spectators’ evaluative attitudes toward the event brand but also by the normative influences exerted by social groups. This dual influence aligns closely with the theoretical assumptions of the Theory of Reasoned Action (TRA), whose dual-mechanism structure makes it uniquely suited for analyzing sports event consumption. In this context, experiential factors simultaneously influence both cognitive evaluations and the formation of social identity. The inherent duality of sports events-combining personal assessment with socially embedded practices-aligns well with the foundational logic of TRA. Competitive aspects engage evaluative processes that shape attitudes, while experiential elements activate identification mechanisms that give rise to normative influences. Based on this theoretical alignment, we propose that sports event brand experience influences spectator loyalty through two distinct pathways: first, by enhancing brand evaluations, thereby strengthening spectators’ attitudes^11^; and second, by shaping social identity, thereby reinforcing subjective norms through social affiliation processes^12^. This dual-pathway mediating mechanism of “attitude–subjective norms” offers a robust framework for examining the relationship between sports event brand experience and spectator loyalty. Nevertheless, this mechanism may exhibit variations due to the heterogeneity of spectator groups, particularly the well-documented influence of gender on sports consumption behavior. Previous research has indicated that men tend to place greater emphasis on the connection between competitive experiences and attitudes, while women are more influenced by social atmospheres and subjective norms^13^. However, it remains unclear whether gender moderates the effects of attitudes and subjective norms on spectator loyalty within the framework of sports event brand experience, and whether the moderating effects differ between the two pathways.

To address these gaps, this study constructs an integrated model incorporating dual mediation pathways and gender-based moderation effects. It seeks to answer three key research questions: First, how does sports event brand experience influence spectator loyalty through the dual pathways of attitudes and subjective norms? Second, what is the relative importance of these two mediating pathways? Third, does gender moderate the strength of influence along these pathways? By answering these questions, this study aims to enhance the theoretical understanding of the mechanisms underlying sports event brand experience and provide evidence-based insights for developing targeted brand management strategies in the sports industry.

## Literature review

### Theoretical foundations

#### Concept definition: event brand experience and sports event brand experience

Brand experience has become a central topic in brand management within the context of the experience economy framework introduced by Pine and Gilmore^14^. This experiential perspective emphasizes the subjective feelings of consumers during brand interactions, shifting focus from functional benefits to memorable experiences. Brakus et al.^15^ define brand experience as “the collection of subjective, internal, and behavioral responses produced by consumers under the stimulation of a brand,” a definition that has provided the foundation for the systematic study of brand experience.

Sports event brand experience (SEBE) represents the application and extension of brand experience in the context of sports events, characterized by its unique social and emotional attributes. Funk and James^3^ highlight that sports event experiences encompass not only attention to the game itself but also the interaction and emotional resonance among spectators. Additionally, the service process of sports events is highly participatory, with spectators functioning as both recipients of services and co-creators of the live atmosphere^16^. Building on these insights, Yoshida and James^16^ define SEBE as “the multidimensional and holistic perception formed by spectators in the process of watching the event,” capturing the comprehensive and interactive nature of the sports event experience.

Early research on SEBE primarily focused on the competitive viewing aspect, often neglecting the emotional and social dimensions. With the advancement of experience marketing theory, Schmitt’s^17^ three-dimensional framework—comprising sensory, emotional, and social dimensions—has been widely adopted in sports event research. The introduction of the service process perspective further expanded this theoretical framework. Parasuraman et al.^18^ proposed a three-dimensional division of interaction quality, environment quality, and outcome quality, which was later applied to sports events by Ko et al.^19^ to reveal the differential impacts of service process components on spectator experiences. Building upon these theoretical foundations, recent research has begun to explore how individual differences-particularly gender-shape spectators’ responses to sports event brand experiences.

Understanding SEBE requires recognition of how it resonates with spectators’ emotions and social identities. Research indicates gender-specific processing patterns, with men typically prioritizing competitive aspects while women respond more strongly to social elements^20,21^. These differences necessitate differentiated brand management approaches to align with diverse spectator expectations.

In addition, value creation theory has provided significant theoretical support for understanding SEBE. Adhikar^22^ emphasized both practical and hedonic aspects of sports event experiences, proposing a dichotomous framework of functional and emotional values. The applicability of this framework has been validated in subsequent studies^23^Recent research by Klaus P P et al.^24^ and Sakuntala, D^25^. has further advanced this field by identifying contextual measurement challenges and demonstrating segment-specific variations in how brand experiences influence consumer behavior.

#### Core model of theory of reasoned behavior

TRA, first proposed by Fishbein and Ajzen^10^posits that behavioral intentions are determined by two key antecedents: ATT and SN. This theoretical framework aligns particularly well with sports event contexts, where consumption occurs within social settings and decisions primarily involve attitudinal and normative considerations rather than significant behavioral control factors. ATT reflect an individual’s positive or negative evaluations of a behavior, shaped by beliefs about outcomes and their value assessments. SN, on the other hand, represent perceived social pressures, including the expectations of significant others and the individual’s motivation to conform. In the context of sports events, ATT is influenced by perceptions of event content, competitive quality, and overall experience, while SN is shaped by group identification and the social atmosphere of the event.

TRA assumes that behavioral intention is the immediate antecedent of actual behavior, with ATT and SN influencing intentions through distinct pathways. Although Ajzen^26^ later extended TRA into the Theory of Planned Behavior (TPB) to account for more complex contexts^27^ the dual-pathway model of TRA remains highly applicable in the collective consumption scenario of sports events. This framework highlights the critical role of SEBE in shaping ATT and SN, which in turn drive SL.

Building on the TRA framework, this study examines how CESE and PESE influence SL through ATT and SN. Specifically, CESE enhances ATT by addressing spectators’ functional and cognitive needs, while PESE strengthens SN by creating an engaging social atmosphere and fostering emotional connections. Furthermore, gender is introduced as a moderating variable, with existing research suggesting that males prioritize functional attributes (e.g., competition and performance), while females place greater emphasis on social and emotional aspects of the event^26^. This study seeks to deepen the application of TRA in the context of sports events and provide theoretical guidance for differentiated brand management strategies.

To enhance our analytical framework, we integrate TRA with two complementary perspectives: the hierarchical brand experience model (distinguishing core competitive elements from peripheral atmospheric elements) and gender-based processing theories (explaining segment-specific pathway variations), It can explain the general process of loyalty formation and the changes in specific market segments, promoting theoretical understanding and the practical application of sports marketing.

#### Research variables

Spectator loyalty (SL) serves as a critical outcome variable of sports event brand experience (SEBE), encompassing two dimensions: attitudinal loyalty and behavioral intentions^28^. In the context of sporting events, SL is expressed not only as the intent to re-attend events but also as emotional attachment to the event brand and positive word-of-mouth recommendations^3^. Clarifying the structure and drivers of SL is crucial for understanding how brand experiences influence loyalty behaviors in the sports event context.

The complexity of SEBE as a multidimensional construct arises from the interaction between spectators and the event brand at multiple levels. This study adopts a two-dimensional framework comprising Core Event Sports Experience (CESE) and Peripheral Event Sports Experience (PESE), which capture the functional and emotional-social values of event experiences, respectively^29^. CESE focuses on the competitive quality and service delivery of the event, directly addressing spectators’ cognitive needs and shaping their evaluations of the event brand. In contrast, PESE emphasizes the atmospheric, social, and emotional dimensions of the event environment, enhancing spectators’ emotional engagement and fostering social interaction. Together, these dimensions provide a comprehensive perspective on SEBE, suggesting its potential to influence SL through distinct cognitive and affective pathways.

To further uncover how these two dimensions of SEBE exert influence, this study draws upon the dual-pathway logic of the Theory of Reasoned Action (TRA). Specifically, it will investigate how CESE and PESE indirectly affect SL via ATT and SN, respectively. CESE is hypothesized to enhance ATT by addressing spectators’ functional and cognitive needs, while PESE is expected to strengthen SN by creating an engaging social atmosphere and fostering emotional connections. By aligning SEBE’s multidimensional nature with the dual conceptual framework of TRA, this study intends to provide new insights into the mechanisms through which brand experience impacts loyalty behaviors in the context of sports events.

### Research hypotheses

#### Influence mechanisms of sport event brand experience

SEBE is a multidimensional construct that reflects spectators’ emotional, cognitive, and behavioral responses to the event brand during consumption^30^. Acting as a crucial vehicle of interaction between event brands and spectators, SEBE not only shapes spectators’ overall perception of the event but also serves as a key driver of loyalty behavior. Brakus, J. J. et al.^15^ demonstrate that multisensory brand experiences create meaningful experiential effects that enhance loyalty through cognitive and affective pathways.

Drawing from TRA, we posit that SEBE influences SL through dual psychological mechanisms-attitudes and subjective norms a framework validated in contemporary sports contexts^31,32^. These mechanisms manifest when brand experiences shape evaluative judgments and perceptions of social expectations. SEBE’s core-peripheral structure aligns with this dual-pathway model, as CESE primarily influences cognitive evaluations while PESE enhances social connections and normative pressures.

##### The role of core and environmental experiences

SEBE is conceptualized as comprising two dimensions CESE and PESE. CESE focuses on the intrinsic aspects of the event, such as the level of competition and professionalism of the content, which directly address spectators’ expectations and cognitive evaluations of the event. In contrast, PESE relates to the emotional and social aspects of the event environment, including the convenience of venue facilities and the quality of service interactions, which enhance spectators’ emotional resonance and social identity with the brand^33^. Recent empirical research by Theodorakis^34^ and Nanu^35^ has empirically validated this dimensional structure, demonstrating that CESE and PESE operate as distinct but interrelated components that collectively shape the overall spectator experience across contemporary consumption environments. Together, these two dimensions are posited to act on spectators’ ATT, shaping their intrinsic evaluations of the event brand. TRA emphasizes that ATT are a key antecedent of behavioral intentions, representing an individual’s positive or negative evaluations of a behavior based on their beliefs and value assessments. Applied to the context of sports events, this suggests that SEBE, through its core and environmental dimensions, may positively influence SL by enhancing ATT toward the event brand. Based on this reasoning, the following hypothesis is proposed:

H1: SEBE has a significant positive effect on spectators’ ATT.

##### The mediating role of attitudes

While ATT serve as a direct determinant of behavioral intentions, they also play a mediating role in linking SEBE to loyalty behavior. Positive ATT toward the event brand not only reflect spectators’ favorable evaluations but also strengthen their emotional attachment and cognitive identification with the brand, fostering long-term loyalty. Research has demonstrated that spectators with favorable ATT are more likely to continue attending events, and to recommend the brand to others^5,36^. Kural et al.^32^ confirm that positive attitudes toward sports events directly predict both behavioral and attitudinal loyalty. This attitudinal loyalty creates a reinforcing feedback loop, where positive ATT enhance both behavioral loyalty and brand equity over time. By integrating SEBE into this framework, it is hypothesized that ATT act as both a direct driver and a mediator of loyalty behavior. Thus, the following hypothesis is proposed:

H2: Spectators’ ATT have a significant positive effect on SL.

##### The role of subjective norms

In addition to ATT, SEBE is hypothesized to influence loyalty behavior through SN, which represent individuals’ perceptions of group expectations and social pressures. TRA posits that SN are a critical determinant of behavioral intentions, especially in scenarios involving collective consumption. The collective nature of sports events creates an ideal context for social interactions, such as discussing the event with friends or experiencing the contagiousness of the on-site atmosphere. These interactions reinforce spectators’ perceptions of group behavioral expectations, making SN a key driver of loyalty behavior^37^. Recent research confirms that social identity derived from sports events enhances susceptibility to normative influences, strengthening loyalty intentions^32^. By facilitating social connections and fostering a sense of shared experience, SEBE enhances spectators’ alignment with the expectations of their reference group, thereby promoting loyalty behaviors. Based on this theoretical foundation, the following hypothesis is proposed:

H3: SEBE has a significant positive effect on SN.

#### Multiple path effects

Within the framework of TRA, an individual’s behavioral intentions are influenced by both internal ATT and external SN. SN, defined as individuals’ perceptions of the expectations of significant others and their motivation to respond to those expectations, serve as a mechanism for perceiving social pressure. In the context of sporting events, the collective consumption experience amplifies the role of SN in shaping loyalty behaviors^23^. For example, when spectators perceive support or expectations from reference groups (e.g., family, friends, or broader social opinion), they are more likely to exhibit loyalty behaviors that align with these expectations, such as consistently attending events or recommending the event brand to others. Based on this reasoning, the following hypothesis is proposed:

H4: SN have a significant positive effect on SL.

Beyond the indirect effects of ATT and SN, SEBE may also exert a direct impact on loyalty behaviors. As a multidimensional construct, SEBE enables spectators to establish a sense of brand belonging and psychological connection during the consumption process through sensory stimulation and emotional resonance^38^. This direct emotional bond can drive spectators to exhibit higher loyalty intentions, even without relying exclusively on the mediating roles of ATT and SN^39^. Such direct effects are particularly pronounced in the immersive consumption scenarios of sporting events, where spectators are deeply engaged with the brand experience. Therefore, the following hypothesis is proposed:

H5: SEBE has a significant direct positive effect on SL.

Finally, ATT not only serve as a direct determinant of loyalty behavior but also indirectly influence loyalty through SN. Positive ATT reflect an individual’s intrinsic recognition of the brand and can reinforce the formation of SN through mechanisms such as social communication and group interaction. Research by Scuotto et al.^40^ demonstrates that when consumers hold positive attitudes toward a brand, they become more attuned to and motivated by the social expectations surrounding that brand. For example, spectators with favorable ATT are more likely to share their event experiences with others or express their support for the brand, thereby enhancing their perception of reference group expectations. This attitude-driven social communication mechanism indirectly drives loyalty behaviors by strengthening SN^41^. Accordingly, the following hypothesis is proposed:

H6: ATT have a significant positive effect on SN.

#### The moderating role of gender

Gender, as a critical social attribute of consumer behavior, shapes consumption preferences and decision-making paths through differences in value pursuits and behavioral tendencies^42^. According to social role theory, men and women develop distinct preferences during the socialization process due to differing role expectations. Men tend to prioritize core functional values, such as utility and performance, whereas women are more focused on emotional experiences and environmental factors^20^. Sport-specific research has substantiated these processing differences. Dietz-Uhler et al.^43^ documented that female consumers integrate emotional and cognitive responses to sports brand elements simultaneously, whereas male consumers evaluate these dimensions sequentially and independently. Similarly, Ren’s^44^ empirical investigation of spectator behavior revealed that women develop loyalty through concurrent processing of personal satisfaction and social identification mechanisms, while men prioritize performance-based assessments before considering social dimension influences. These differences suggest that gender may play a moderating role in the pathways linking SEBE, ATT, SN, and SL.

First, gender may moderate the effect of brand experience on ATT (H7a). SEBE encompasses both CESE, which reflects the level of competition and professionalism, and PESE, which includes service quality and event atmosphere. Men are more likely to develop stronger attitudinal responses when the brand experience emphasizes professionalism and competitiveness, as they prioritize core functional attributes. Women, on the other hand, are more sensitive to emotional and social aspects of the experience, such as venue atmosphere and interpersonal interactions, which may lead to greater attitudinal enhancement via emotional resonance. Recent evidence reveals significant gender-based variations in attitudinal formation processes. Women demonstrate more holistic, emotionally-integrated cognitive structures with stronger cross-loading between affective and cognitive components, while men exhibit more compartmentalized, sequential evaluation patterns^45^.

Second, gender may moderate the relationship between ATT and SN (H7b). ATT, as the cumulative result of affective and cognitive evaluations, are more likely to trigger social interactions among women, thereby reinforcing SN. Women are generally more sensitive to social expectations and group dynamics, leading to a stronger transmission of SN through attitudinal pathways. In contrast, men rely more on intrinsic motivation and personal judgment, which may result in a weaker influence of ATT on SN.

Third, gender may influence the direct relationship between brand experience and SN (H7c). In the context of group consumption, women are more likely to perceive enhanced group expectations through brand experiences, particularly when these experiences involve social or cultural elements. For instance, the social atmosphere of an event or the cultural values promoted by a brand may resonate more strongly with women, amplifying their perception of SN. Men, by comparison, tend to exhibit lower sensitivity to social pressure, leading to a relatively weaker direct effect of brand experience on SN^46^.

Additionally, gender differences may affect the relationship between SN and loyalty behaviors (H7d). Women, who are more influenced by social expectations, are likely to exhibit stronger loyalty behaviors when SN align with group expectations. Conversely, men are more driven by personal ATT and internal perceptions, which diminishes the relative impact of SN on their loyalty behaviors^38^.

Finally, gender may moderate the direct effects of brand experience and ATT on loyalty behaviors (H7e and H7f). Men, with their focus on core event attributes such as competition and professionalism, are more likely to demonstrate loyalty behaviors directly driven by SEBE and positive ATT. For instance, highly competitive events are particularly effective in fostering loyalty among male spectators. Women, on the other hand, are more influenced by emotional resonance and indirect pathways, such as SN, to enhance their loyalty behaviors. This indicates that the strength of the direct and indirect pathways from SEBE and ATT to loyalty behaviors varies by gender.

Based on the above analysis, the following hypotheses are proposed:

H7a: Gender moderates the effect of SEBE on ATT.

H7b: Gender moderates the relationship between ATT and SN.

H7c: Gender moderates the effect of brand experience on SN.

H7d: Gender moderates the relationship between SN and SL.

H7e: Gender moderates the direct effect of SEBE on SL.

H7f: Gender moderates the direct effect of ATT on SL.

**Fig. 1:**
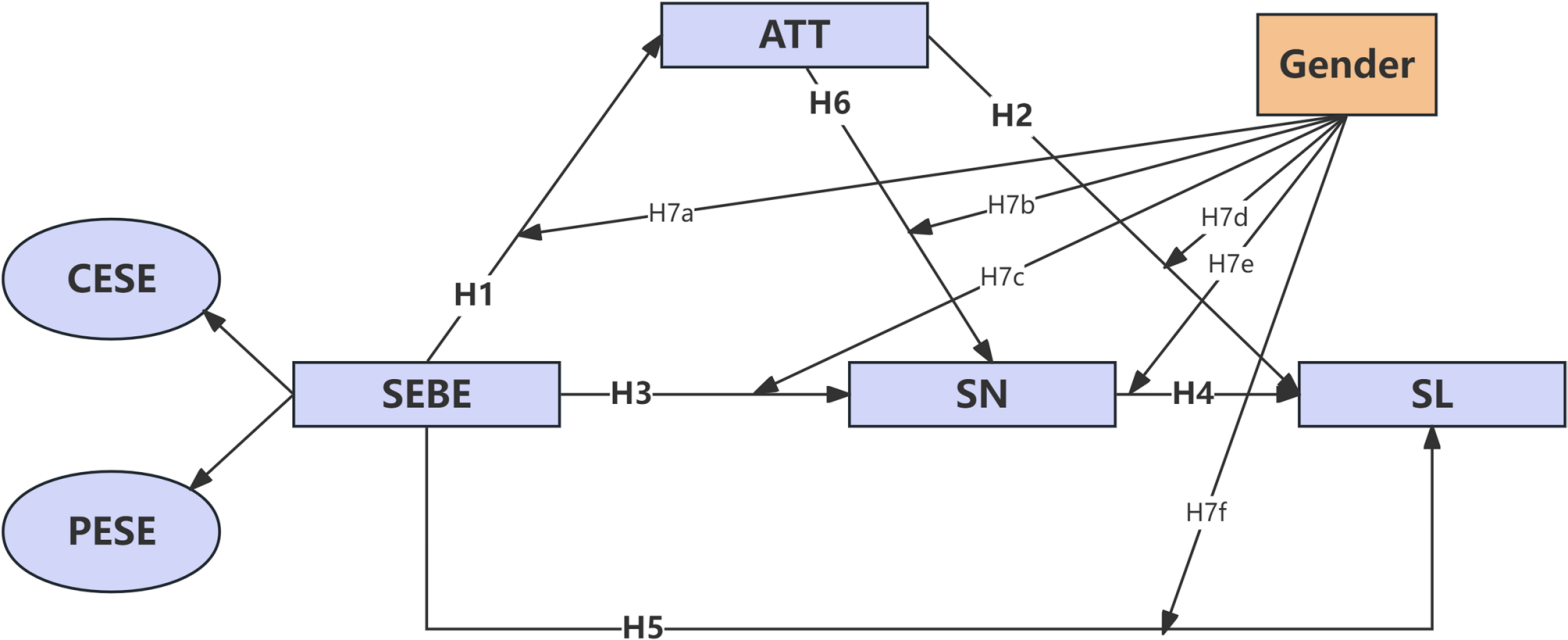
Model of the influence of brand experience of sports events on audience loyalty. Note: CESE: Core Event Experience, PESE: Peripheral Event Experience, SEBE: Sports Event Brand Experience, ATT: Attitude, SN: Subjective Norm, SL: Spectator Loyalty.

In summary, this study proposed a framework of hypotheses to explore the effects of SEBE on SL (see Fig. 1), including direct effects, mediating roles of ATT and SN, and the moderating influence of gender. This framework integrates contemporary research on brand experiences, consumer attitudes, and gender differences in sports consumption to provide a comprehensive theoretical basis for understanding loyalty formation mechanisms.

## Research design

### Research objects

China’s booming sports market, driven by large-scale events such as the Winter Olympics, Asian Games, and World University Games, has attracted a diverse audience across regions, genders, and age groups. This provides an ideal context for examining the diversity and universality of SEBE. To comprehensively understand Chinese spectators’ perceptions of SEBE and their loyalty behaviors, this study adopted a stratified random sampling method.

The stratified random sampling procedure was executed via the Wenjuanxing online survey platform. The sampling frame was partitioned into mutually exclusive strata based on two primary dimensions: event typology (team sports, individual sports, and multi-sport events) and geographical distribution. This stratification ensured heterogeneity between strata while maintaining homogeneity within each stratum. Random selection was then applied within each defined stratum, maintaining probabilistic sampling principles. A qualifying criterion requiring respondents to have personally attended sports events more than once in the previous twelve months was implemented to ensure respondent relevance to the research objectives. This methodological approach optimized sample representativeness across the diverse Chinese sports spectator population while preserving the statistical advantages of randomization.

The final valid sample demonstrates high representativeness, encompassing respondents of varying genders, age groups, educational levels, and viewing frequencies (see Table 1 for detailed demographic statistics). Among the respondents, 69.177% were male and 30.823% were female, with an age range spanning from under 18 to over 46 years old. The largest group of viewers was aged 18–25 (26.858%). Regarding educational background, 49.157% of respondents held a bachelor’s degree or higher. Viewing frequency data revealed that 52.131% of respondents attended sports events one to three times in the past year, while 36.670% reported attending events seven times or more annually. This balanced sample distribution and extensive demographic coverage provide comprehensive and reliable data for subsequent analysis of SEBE and its associated variables.

**Table 1:** Distribution of basic characteristics of the respondents.

Category of features	Grouping	Frequency(*N*)	Percentage(%)
Gender	Male	698	69.177
	Female	311	30.823
Age	Under 18 years of age	141	13.974
	18–25 years old	271	26.858
	26–35 years old	248	24.579
	36 to 45 years old	189	18.731
	46 years of age and older	160	15.857
Level of education	High school/technical secondary school or below	268	26.561
	Junior college	245	24.281
	Undergraduate	373	36.967
	Master’s degree or above	123	12.190
Frequency of viewing	1 to 3 times	526	52.131
	4–6 times	113	11.199
	7 times or more	370	36.670

### Questionnaire design

To ensure the reliability and validity of the questionnaire, this study adopted proven and established scales from prior research. Specifically, it incorporated measures of event quality and venue environment^37^ a service experience scale^18^ an interactive experience assessment^47^ models for measuring affective dispositions and cognitive appraisals^48,49^ the Theory of Planned Behavior scale^26^ and loyalty measures^38,50^. The questionnaire was designed to measure four core variables—SEBE, ATT, SN, and SL (see Table 2 for detailed measurement items and sources). Each variable was defined and operationalized as follows: (1) SEBE: Divided into two dimensions—CESE, focusing on the event’s intrinsic aspects, and PESE, addressing environmental and supplementary elements. (2) Attitude (ATT): Consisting of two dimensions—affective tendencies and cognitive evaluations. (3) SN: Including two dimensions—perceptions of social influence and social evaluation. (4) SL: Incorporating two dimensions—behavioral loyalty and attitudinal loyalty. A total of 34 items were refined through exploratory factor analysis to ensure construct validity. To minimize common method bias, the questionnaire items were randomly arranged during the survey process. The final questionnaire was systematically structured to examine the relationships among SEBE, ATT, SN, and SL while maintaining high reliability and validity.

**Table 2:** Measurement indicators and sources of each variable.

Variables of interest	Indicators	Content	Source
Sports event brand experience	Event quality experience	The degree of excellence of the game, the intensity of competition, the professionalism of the referee’s law enforcement, etc.	(Smith, A. C. & Stewart, B., 2010)
	Organizational service experience	The attitude and efficiency of the service personnel, the timeliness of the event information, etc.	( Parasuraman et al., 1988 )
	Venue environment experience	Seat comfort, venue convenience and safety, etc.	(Smith, A. C. & Stewart, B., 2010)
	Interactive experience	The enthusiasm of the audience to participate in the activity, the interactive experience with other audiences, etc.	(Yi, Y. & Gong, T., 2013)
Attitude	Tendency to affect	The degree of love and sense of belonging to the game	(Keller, K. L., 1993)
	Assessment of cognition	Rational cognition of the image and professionalism of the tournament brand	(Fishbein, M., & Ajzen, I., 1975)
Subjective Norm	Perception of social influence	The attitudes of family and friends to the tournament brand and its influence	(Ajzen, I., 1991)
	Cognition of social evaluation	Social and cultural recognition of the event, the social reputation of the event brand, etc.	(Bandura, A., 1986)
Spectator Loyalty	Loyalty of behavior	The possibility of continuing to watch the game, the willingness to purchase the game related products or services, and the recommendation behavior	(Oliver, R. L., 1999)
	Loyalty of attitude	The degree of trust in the competition brand and the willingness to consistently support that brand over the long term	(Reichheld, F. F. & Sasser, W. E., 1990)

### Questionnaire distribution and collection

Before the formal release of the questionnaire, a pre-survey was conducted using a stratified sampling method with 430 respondents to assess its reliability and validity. The pre-survey results demonstrated high internal consistency and structural validity, confirming that the questionnaire was logically designed.

The formal survey was conducted through the Questionnaire Star platform from August 5, 2024, to December 3, 2024. A total of 1,040 responses were collected, of which 1,009 valid questionnaires were retained after excluding invalid responses (e.g., extreme answers or excessively short completion times), resulting in a validity rate of 97.02%. This sample size exceeded the minimum requirement for structural equation modeling (SEM) analysis^51^. Ethics approval was obtained from the Ethics Committee of the School of Nursing and Rehabilitation, Shandong University (Approval No. 2021-R-001). All procedures were conducted in accordance with the Declaration of Helsinki, with voluntary participation, informed consent, and the option to withdraw at any time. Data were collected anonymously, kept confidential, and posed no risks to participants.

### Data analysis

The data analysis was conducted in five stages: (1) Descriptive Statistical Analysis: SPSS 26.0 was used to analyze respondents’ basic demographic characteristics, such as gender and age. (2) Reliability Testing: Cronbach’s alpha coefficient was used to assess the internal consistency of the scales. (3) Validity Testing: This included assessments of convergent and discriminant validity. The Kaiser-Meyer-Olkin (KMO) test and Bartlett’s sphericity test were conducted to ensure the data’s suitability for factor analysis. (4) Exploratory and Confirmatory Factor Analysis: Exploratory Factor Analysis (EFA) was conducted to evaluate construct validity, followed by Confirmatory Factor Analysis (CFA) to assess the model’s fit. (5) Structural Equation Modeling (SEM): SmartPLS 4.0 was used to build and analyze the SEM, exploring the relationships among SEBE, ATT, SN, and SL, and to test the research hypotheses and mediating effects.

## Findings of the study

### Common method bias

To assess potential common method bias, Harman’s single-factor test was conducted. Unrotated factor analysis extracted factors with eigenvalues greater than 1, and the results showed that the first factor explained 31.048% of the variance, which is below the critical threshold of 40%. The cumulative explained variance contribution was 77.477%, with multiple factors extracted, indicating no significant common method bias. These results confirm the suitability of the data for subsequent analyses.

### Exploratory factor analysis

Exploratory Factor Analysis (EFA) was conducted using SPSS 26.0 with principal component analysis and Kaiser-normalized maximum variance rotation as the rotation method^52^. Items with factor loadings below 0.60 were excluded, resulting in the retention of 29 valid items. The factor loadings for all dimensions ranged from 0.614 to 0.843, meeting the criteria for model construction. The results were statistically significant, confirming the construct validity of the variables.

### Measurement model analysis

#### Reliability and validity of first-order latent variable models

The reliability and validity of all first-order latent variables were assessed (see Table 3). The results showed that all factor loadings exceeded the threshold of 0.7, indicating strong associations between the measures and their respective latent variables. Cronbach’s alpha coefficients and composite reliability (CR) values were above 0.8, confirming the internal consistency of the measures. Furthermore, the average variance extracted (AVE) values for all constructs exceeded 0.6, demonstrating satisfactory convergent validity. These findings validate the measurement model’s reliability and provide a solid foundation for subsequent analyses of second-order latent variables and structural modeling.

**Table 3:** Evaluation of the first-order latent variable measurement model.

First order latent variable	Explicit variable	Factor loading	Cronbach’s α	CR	AVE
ATT	ATT1	0.833	0.916	0.918	0.704
	ATT2	0.838			
	ATT3	0.836			
	ATT4	0.848			
	ATT5	0.839			
	ATT6	0.842			
SEBE	SEBE1	0.813	0.891	0.892	0.648
	SEBE11	0.787			
	SEBE3	0.792			
	SEBE5	0.787			
	SEBE7	0.813			
	SEBE9	0.837			
	SEBE10	0.830	0.867	0.869	0.654
	SEBE2	0.818			
	SEBE4	0.837			
	SEBE6	0.717			
	SEBE8	0.834			
SL	SL1	0.852	0.928	0.929	0.735
	SL2	0.861			
	SL3	0.856			
	SL4	0.863			
	SL5	0.854			
	SL6	0.857			
SN	SN1	0.797	0.886	0.887	0.638
	SN2	0.792			
	SN3	0.802			
	SN4	0.808			
	SN5	0.791			
	SN6	0.801			

Note: SEBE.Sports Event Brand Experience; ATT.Attitude SN.Subjective Norm SL.Spectator Loyalty.

#### Reliability and validity analysis of the second-order latent variable model

The reliability and validity of the second-order latent variables were assessed (see Table 4). The results showed that the composite reliability (CR) values for all second-order latent variables exceeded the threshold of 0.8, and the average variance extracted (AVE) values were greater than 0.6. Additionally, the square root of the AVE for each latent variable was higher than the Pearson correlation coefficients between the other second-order latent variables, confirming satisfactory discriminant validity. These findings demonstrate that the second-order latent variable model possesses robust reliability and validity, providing a strong foundation for the subsequent structural equation modeling (SEM) analysis.

**Table 4:** Reliability and validity analysis of the second-order latent variable measurement model.

Second-order latent variable	CR	AVE	ATT	SEBE	SL	SN
ATT	0.918	0.704	0.839			
SEBE	0.693	0.765	0.192	0.875		
SL	0.929	0.735	0.169	0.461	0.857	
SN	0.887	0.638	0.291	0.318	0.444	0.798

Note: SEBE.Sports Event Brand Experience; ATT.Attitude SN.Subjective Norm SL.Spectator Loyalty.

### Structural equation modeling analysis

#### Structural equation modeling path analysis

The results of the structural equation modeling (SEM) path analysis confirmed the hypothesized relationships among the variables (see Table 5). Specifically, SEBE had a significant direct positive effect on SL (β = 0.355, *p* < 0.001). SEBE also significantly influenced Attitude (ATT) (β = 0.192, *p* < 0.001) and SN (β = 0.272, *p* < 0.001). Furthermore, ATT had a significant positive effect on SN (β = 0.239, *p* < 0.001), and SN significantly influenced SL (β = 0.330, *p* < 0.001). These results indicate that SEBE not only directly affects SL but also indirectly influences SL through ATT and SN, highlighting the mediating roles of ATT and SN in the process.

**Table 5:** Structural model analysis.

	The path	β	STDEV	T	*P* values	Results
DE	ATT-> SN	0.239	0.032	7.459	0.000	Set up
	SEBE-> ATT	0.192	0.033	5.826	0.000	Set up
	SEBE-> SL	0.355	0.031	11.358	0.000	Set up
	SEBE-> SN	0.272	0.031	8.812	0.000	Set up
	SN-> SL	0.330	0.031	10.479	0.000	Set up
	SEBE-> SN-> SL	0.090	0.013	7.160	0.000	Set up
IE	SEBE-> ATT-> SN-> SL	0.015	0.004	4.025	0.000	Set up
	SEBE-> ATT-> SL	0.001	0.006	0.132	0.895	Not true
	SEBE-> ATT-> SN	0.046	0.010	4.781	0.000	Set up
	ATT-> SN-> SL	0.079	0.014	5.732	0.000	Set up
	ATT-> SL	0.079	0.014	5.732	0.000	Set up
	SEBE-> SL	0.106	0.014	7.808	0.000	Set up
	SEBE-> SN	0.046	0.010	4.781	0.000	Set up

Note: SEBE.Sports Event Brand Experience; ATT.Attitude SN.Subjective Norm SL.Spectator Loyalty.

The analysis further validated the study’s hypotheses (H1–H6): H1: SEBE has a significant positive effect on ATT (β = 0.192, *p* < 0.001); H2: ATT significantly influences SL (β = 0.079, *p* < 0.001); H3: SEBE has a significant positive effect on SN (β = 0.272, *p* < 0.001); H4: SN significantly influences SL (β = 0.330, *p* < 0.001).

H5: SEBE directly influences SL (β = 0.355, *p* < 0.001); H6: ATT significantly influences SN (β = 0.239, *p* < 0.001).

Overall, all path coefficients were statistically significant at the 0.001 level, confirming a strong causal relationship among SEBE, ATT, SN, and SL. The results also verified the rationality of the theoretical framework proposed in this study.

#### Mediated effects test

Following the recommendations of prior research^53^ this study employed the BC-Bootstrap method to test for mediated effects. The results indicated that the indirect effect of SEBE on SL through SN was significant (β = 0.090, *p* < 0.001), and the chain-mediated effect of SEBE on SL via Attitude (ATT) and SN was also significant (β = 0.015, *p* < 0.001). However, the indirect effect of SEBE on SL mediated solely by ATT was not significant (β = 0.001, *p* = 0.895). Additionally, the indirect effect of ATT on SL through SN was significant (β = 0.079, *p* < 0.001), and the total indirect effect of SEBE on SL was significant (β = 0.106, *p* < 0.001). These results highlight the critical mediating role of SN in the relationship between SEBE and SL. Although ATT has a direct effect on SL (β = 0.083, *p* = 0.008), its primary influence is exerted indirectly through SN. In summary, the mechanism by which SEBE influences SL is dominated by its direct effect, with significant indirect effects mediated through SN and the ATT-SN chain. These findings provide deeper insights into the complex relationships among SEBE, ATT, SN, and SL.

#### Evaluation of structural equation modeling

Based on established recommendations^54^ this study evaluated the structural model using the coefficient of determination (R²), the effect size (f²), and the predictive relevance (Q²) (see Table 6). The R² value of SL was 0.311, indicating that 31.1% of the variance in SL was explained by the model, reflecting a moderate level of explanatory power. The R² values for SN and ATT were 0.156 and 0.037, respectively, indicating explanatory power levels of 15.6% and 3.7%. In terms of effect size (f²), the effect of SEBE on SL was moderate (f² = 0.162), as was the effect of SN on SL (f² = 0.133). The effects of SEBE on SN (f² = 0.085) and ATT (f² = 0.038) were small to moderate, while the effect of ATT on SN (f² = 0.065) was also small to moderate. However, the effect of ATT on SL was negligible (f² ≈ 0).

**Table 6:** Structural model evaluation.

Structural model	β	Standard deviation (STDEV)	T	*P*	f^2^	VIF	*R* ^2^	Q^2^
ATT-> SL	0.004	0.033	0.134	0.894	0.000	1.106	0.311	0.226
SEBE-> SL	0.355	0.031	11.358	0.000	0.162	1.126		
SN-> SL	0.330	0.031	10.479	0.000	0.133	1.185		
ATT-> SN	0.239	0.032	7.459	0.000	0.065	1.038	0.156	0.099
SEBE-> SN	0.272	0.031	8.812	0.000	0.085	1.038		
SEBE-> ATT	0.192	0.033	5.826	0.000	0.038	1	0.037	0.025

Note: SEBE.Sports Event Brand Experience; ATT.Attitude SN.Subjective Norm SL.Spectator Loyalty.

In assessing predictive relevance (Q²), the results showed that the Q² of SL was 0.226, indicating a strong predictive ability. The Q² values for SN and ATT were 0.099 and 0.025, respectively, suggesting that the structural model had predictive relevance at varying levels. Additionally, the variance inflation factor (VIF) values for all variables ranged between 1.000 and 1.185, which were well below the threshold of 3.3, confirming that the model did not suffer from serious multicollinearity issues and that there was good differentiation among the variables. In summary, the structural model demonstrated robust explanatory and predictive power, particularly in explaining SL. The effects of SEBE and SN on SL were more significant, while the influence on ATT was relatively weak. These results underscore the central roles of SEBE and SN in shaping SL.

### Multi-cohort analysis

**Fig. 2:**
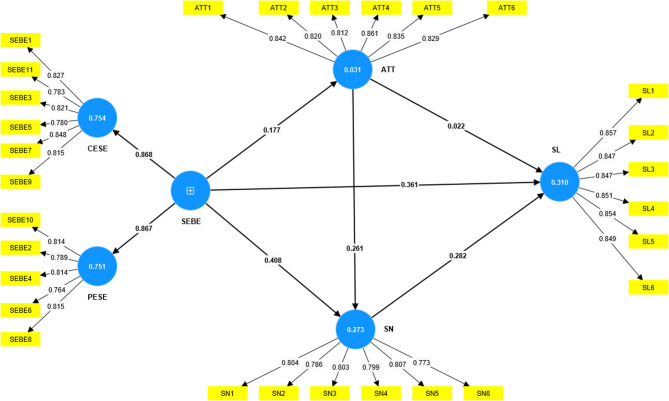
Structural equation model test results for female viewers.

**Fig. 3:**
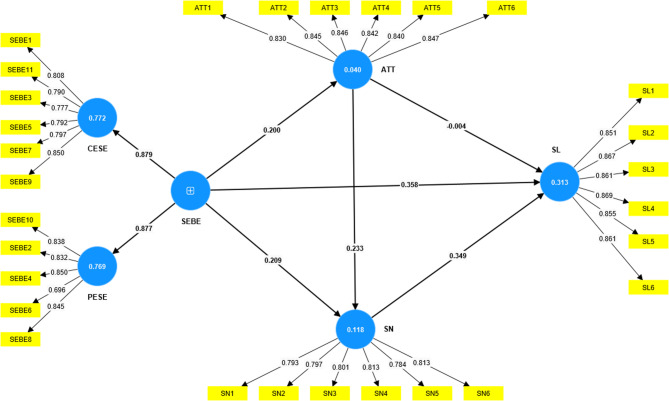
Structural equation model test results for male viewers.

This study employed the PLS-MGA method^55^ to conduct a multi-cohort analysis based on gender, examining the moderating role of gender in the relationships among SEBE, ATT, SN, and SL (see Figs. 2 and 3).

The results of the multi-cohort analysis (Table 7) indicated that most path coefficients were not significantly different between male and female groups. Specifically, the direct effect of SEBE on SL (females: β = 0.361, STDEV = 0.060; males: β = 0.358, STDEV = 0.035; difference = 0.003, *p* = 0.959), the effect of SEBE on ATT (females:β = 0.177, STDEV = 0.057; males: β = 0.200, STDEV = 0.041; difference = −0.023, *p* = 0.741), the effect of ATT on SN (females: β = 0.261, STDEV = 0.053; males: β = 0.233, STDEV = 0.038; difference = 0.028, *p* = 0.669), and the effect of SN on SL (females: β = 0.282, STDEV = 0.061; males: β = 0.349, STDEV = 0.037; difference= −0.067, *p* = 0.350) did not show significant differences between genders. Similarly, the effect of ATT on SL was minimal and not significantly different for both groups (difference = 0.026, *p* = 0.711).

**Table 7:** Results of multicluster analysis by gender.

Relationships	Female		Male		Difference sig.test	
	β	STDEV	Original sample	STDEV	Difference	2-tailed *p* value
ATT-> SL	0.022	0.057	−0.004	0.041	0.026	0.711
ATT-> SN	0.261	0.053	0.233	0.038	0.028	0.669
SEBE-> ATT	0.177	0.057	0.200	0.041	−0.023	0.741
SEBE-> SL	0.361	0.060	0.358	0.035	0.003	0.959
SEBE-> SN	0.408	0.053	0.209	0.037	0.199	0.001
SN-> SL	0.282	0.061	0.349	0.037	−0.067	0.350

Note: SEBE.Sports Event Brand Experience; ATT.Attitude SN.Subjective Norm SL.Spectator Loyalty.

However, a significant gender difference was observed in the path linking SEBE to SN. The female group (β = 0.408, STDEV = 0.053) exhibited a stronger effect compared to the male group (β = 0.209, STDEV = 0.037), with the difference being statistically significant (difference = 0.199, *p* = 0.001). This finding suggests that SEBE has a greater impact on SN for females than for males.

In summary, the moderating effect of gender was primarily reflected in the relationship between SEBE and SN, where females were more strongly influenced by SEBE in shaping their SN. Although most paths did not exhibit significant gender differences, these findings provide valuable insights for event organizers. Specifically, marketing strategies targeting female spectators should emphasize social elements and group interactions to enhance their perceptions of SN and, consequently, their loyalty (SL). By addressing the social needs of female spectators, event organizers can strengthen their engagement and foster long-term loyalty.

### Model robustness analysis

#### Non-linear analysis

To test the robustness of the model, a quadratic term was introduced following previous recommendations^56^ to evaluate potential nonlinear effects among SEBE, ATT, SN, and SL. The analysis results indicated that all nonlinear effects were statistically insignificant, suggesting that the relationships among the variables were predominantly linear. Consequently, there was no need to incorporate more complex nonlinear models, confirming the adequacy of the linear modeling approach adopted in this study.

#### Endogeneity analysis

To further ensure the robustness of the model, the Gaussian coupling method^57^ was used to test for potential endogeneity and examine whether reverse causality or interaction effects existed among the variables. The results showed that none of the paths exhibited statistical significance in terms of endogeneity^58^ indicating that the relationships among variables in the model were primarily unidirectional and free from interference caused by endogeneity issues. These findings confirm the reliability of the study’s results and the robustness of the model.

#### Analysis of model heterogeneity

Unobserved heterogeneity was examined using the FIMIX-PLS method^56^ to assess whether latent heterogeneity existed in the dataset. The theoretical maximum number of segments was estimated based on the minimum sample size required per segment, which was calculated to be 138 at an effect size of 0.15 and a statistical power of 80%. Given a total sample size of 1,009, up to 7 segments could theoretically be extracted (1,009 ÷ 138 ≈ 7.31). However, to avoid over-segmentation and maintain the interpretability of the model, FIMIX-PLS analyses were conducted with 1 to 4 segments, following established practice^59^.

In summary, the results of the robustness analysis demonstrated that the relationships among SEBE, ATT, SN, and SL were predominantly linear, free from endogeneity issues, and unaffected by unobserved heterogeneity. These findings validate the reliability of the structural model and provide strong support for the study’s conclusions.

## Discussion, conclusion, and implications

### Discussion and conclusion

Grounded in the TRA, this study systematically examined the mechanisms through which SEBE influences SL. Using multilevel path analysis, the findings revealed the differential impacts of direct effects, indirect effects, and gender-moderated effects. The results not only validate the theoretical proposition that brand experience serves as a core driver of SL but also underscore the critical mediating role of SN in the specific context of sports event consumption, as well as the moderating role of gender in pathway selection. The following sections provide a detailed discussion of the main findings and their theoretical and practical implications.

#### The path of influence of sports event brand experience on spectator loyalty

##### Direct effect: the core role of sports event brand experience in spectator loyalty

This study demonstrates that SEBE exerts a significant direct effect on SL (β = 0.355, *p* < 0.001), confirming prior findings that brand experience is a pivotal driver of loyalty^60,61^. Specifically, CESE provides sensory stimulation and emotional resonance, enhancing brand identity through high-quality competitive content and professional event design^62^. In parallel, PESE complements the core experience by optimizing venue facilities and fostering service interactions, further amplifying loyalty^63^.

The unique consumption scenario of sports events further reinforces the direct effects of brand experience. On the one hand, the highly experiential and contextual nature of events enables spectators to internalize brand values quickly through emotional engagement and on-site immersion^64^. On the other hand, the social and interactive dimensions of sports events magnify the impact of brand experience by fostering group identification and shared interactions^65^. These attributes firmly establish brand experience as a cornerstone of loyalty formation^66^.

Although the indirect effect (β = 0.106) is relatively small, its influence through social interaction mechanisms is significant and cannot be overlooked. This finding aligns with social constructiveness theory^67^ and extends research on multiple motivations for sports consumption^3^ by offering new insights into the dynamic processes underlying loyalty formation. Unlike TRA-based models suggesting threshold effects in experiential contexts^68^ our findings reveal a consistent linear relationship between SEBE and loyalty, reflecting the sustained influence of experiential factors in socially shared sports environments. Furthermore, differences in the contributions of CESE and PESE to loyalty were observed. CESE primarily enhances perceived value and satisfaction, while PESE strengthens emotional bonds through improved service and interaction quality. The synergy between these dimensions explains the moderate effect size of SEBE on SL (f² = 0.162) and highlights their complementary roles.

##### Indirect effects: dual-mediated transmission paths

The findings reveal that the indirect effect of brand experience on loyalty via Attitude (ATT) was insignificant (β = 0.001, *p* = 0.895). This suggests that brand experience alone is insufficient to drive loyalty through attitude mediation, contrasting with the traditional TRA perspective, which views attitude as a key determinant of behavioral intention^26,69^. In the context of sports events, loyalty behaviors appear to be more influenced by external factors, such as group interactions and social norms, rather than intrinsic attitudinal mechanisms^70,71^. This finding aligns with studies highlighting the prominence of scenario-based social influences in shaping consumer behavior during interactive and immediate consumption experiences^72^.

In contrast, SN played a mediating role in the relationship between SEBE and SL (β = 0.090, *p* < 0.001). High-quality event experiences translate into loyalty through enhanced group identity and social expectations. For instance, positive event experiences foster spectators’ sense of belonging and identity via word-of-mouth communication and shared group norms, thereby driving loyalty^73,74^. The significant mediating effect of SN merits deeper analysis, particularly given its stronger influence (β = 0.330, *p* < 0.001) compared to the direct effect of ATT on SL (β = 0.079, *p* < 0.001). This finding reveals the primacy of social mechanisms in collective consumption contexts, where normative influences significantly outweigh individual attitudinal processes in driving loyalty. The gender-differentiated impact of SN further substantiates this interpretation, consistent with findings that social identification exerts stronger effects among demographic groups with heightened sensitivity to collective identity signals^32^. This underscores the dual role of SN as both a transformative mechanism for brand experience and a reflection of the inherently social nature of sports event consumption^75,76^.

Additionally, the chain mediation effect revealed a synergistic interaction between ATT and SN (β = 0.015, *p* < 0.001). While the mediating effect of ATT alone was limited, the influence of SEBE on SL was significantly amplified when SN were incorporated into the transmission mechanism. This finding suggests that in group-based consumption scenarios, individual positive ATT must be reinforced by social norms to effectively translate into loyalty behaviors^77,78^. This chain mediation enriches the theoretical application of TRA by offering novel insights into the multilevel pathways through which brand experience influences loyalty.

##### Moderating effects: patterns of influence of gender differences

This study highlights the moderating effect of gender in the process of transforming SEBE into SL at sporting events, revealing notable differences in path selection between male and female viewers. These differences reflect distinct psychological mechanisms, with social norms playing a particularly important role among female spectators^20^.

First, the direct path from brand experience to loyalty (SEBE-SL) exhibited a high degree of consistency between male and female viewers (female: β = 0.361, male: β = 0.358, *p* = 0.959). This finding indicates that the mechanism by which high-quality event content and environmental experiences drive loyalty through sensory stimulation and emotional resonance is broadly applicable across genders. It also challenges traditional sports marketing assumptions that female viewers are less responsive to event brand experiences. Instead, the results confirm that women, like men, can effectively translate brand experiences into loyalty through the direct pathway, providing support for the development of inclusive marketing strategies.

In contrast, the indirect path (SEBE-SN-SL) through which brand experience influences loyalty via SN revealed significant gender differences (female: β = 0.408, male: β = 0.209, *p* = 0.001). Female viewers were found to rely more heavily on social norms to transform brand experience into loyalty behaviors, whereas male viewers tended to rely more on the direct path^41,79^. Interpreted through social role theory^80^ these findings reflect gender-specific socialization processes in which women typically develop greater sensitivity to interpersonal relationships and group dynamics, while men prioritize achievement and individual outcomes^20^. The stronger SEBE-SN path coefficient among female spectators thus reflects their heightened sensitivity to social identity in sports consumption contexts. High-quality tournament experiences further enhance female spectators’ loyalty by reinforcing their perceptions of reference group expectations. These findings contribute to a deeper understanding of gender differences in sports consumption and highlight the role of social norms in shaping female loyalty^81^.

Further analysis revealed that loyalty formation among female spectators activates both direct and indirect pathways, resulting in a synergistic mechanism that reinforces loyalty through a combination of individual experiences and social interaction^82^. In contrast, male viewers rely predominantly on the direct pathway, leading to a relatively streamlined loyalty formation process^83^. This dual-pathway mechanism enhances the robustness of female loyalty and underscores the potential for community-driven engagement strategies. These insights provide a foundation for more tailored marketing approaches targeting female spectators^84^.

Moreover, the gender-differentiated path selection appears to be context-dependent. The direct path tends to dominate in highly competitive events, while the indirect path becomes more influential in events emphasizing social interaction or entertainment^85,86^. This dynamic interplay between event type and gender characteristics underscores the importance of context-sensitive marketing strategies. For competitive events, efforts should focus on highlighting event content to attract male viewers. Conversely, for socially oriented events, marketers can strengthen female viewers’ perceived social norms through community activities and interactive experiences^87^.

### Theoretical contributions

This study makes important contributions in three key areas: brand experience theory, loyalty formation mechanisms, and gender difference theory. It expands the scope of existing theories and provides new perspectives for understanding consumer behavior in the context of sports events.

First, this study constructs a two-level brand experience framework based on the “core-support” structure, which broadens the dimensions and applicability of traditional brand experience theories. Previous studies have often regarded brand experience as a unified concept or primarily focused on its relationship with service quality^88–90^. While prior research has established conceptual foundations through psychological experience taxonomies and domain-specific classifications, our study advances theoretical development by demonstrating how experience components function as an integrated system with distinct yet interdependent pathways to loyalty. By drawing on service hierarchy theory, this study divides SEBE into CESE and PESE, revealing their differentiated roles in loyalty formation. The findings indicate that core experiences significantly influence loyalty through the direct path (β = 0.412, *p* < 0.001), while peripheral experiences indirectly enhance loyalty intentions by optimizing supportive experiences (β = 0.376, *p* < 0.001). This framework advances brand experience theory by revealing the complementary and synergistic effects of core and peripheral experiences, providing theoretical innovations for studying highly contextualized consumption scenarios.

Second, this study uncovers the multi-path influence of brand experience on loyalty, thereby enriching the theoretical understanding of the “attitude-behavior” relationship and proposing a unique pathway model in the context of sports events. While traditional theories emphasize that attitude serves as a central variable in predicting behavior^69,91,92^this study found that the mediating role of attitude alone was not significant in the context of sporting events (β = 0.001, *p* = 0.895). Instead, brand experience influences loyalty through three primary mechanisms: the direct effect (β = 0.355, *p* < 0.001), the mediating effect of SN (β = 0.090, *p* < 0.001), and the chain mediating effect of “attitude-SN” (β = 0.015, *p* < 0.001). SN play a key role as a social influence mechanism, highlighting how brand experience drives loyalty behavior by enhancing perceptions of group expectations and social identity. Additionally, the efficacy of ATT indirectly influences loyalty by amplifying the role of social norms^93^. These findings extend the applicability of the attitude-behavior theory by emphasizing the importance of group norms and social interactions in sports event consumption.

Finally, this study systematically examines the moderating role of gender, deepening the application of gender difference theory in the field of sports consumption. Existing research has primarily focused on gender differences in participation motivation and consumption preferences^94,95^ but has rarely explored their roles in the mechanisms linking brand experience to loyalty. This study found that gender differences primarily manifest in the “brand experience-SN” pathway (SEBE-SN) (β = 0.199, *p* = 0.001). Female viewers are more sensitive to social norms and are therefore more likely to translate brand experiences into loyalty behaviors through group identification. This finding provides insights into the psychological mechanisms underlying gender differences in socialized consumption decision-making. It also offers a theoretical foundation for precision marketing strategies, emphasizing that female viewers’ loyalty to tournament brands can be effectively enhanced by fostering perceptions of community interaction and social identification.

In summary, this study enriches brand experience theory, the attitude-behavior theory, and gender difference theory by constructing a two-level brand experience framework, revealing the multi-path influence of brand experience on loyalty, and identifying the moderating role of gender. These contributions offer new perspectives and actionable insights for understanding and managing consumer behavior in the context of sports events.

### Management implications

This study provides practical insights for sports event brand management and tourism promotion by emphasizing the integrated use of brand experience optimization, social influence mechanisms, and data-driven marketing strategies. These approaches aim to enhance both the market competitiveness of event brands and audience loyalty.

First, the findings demonstrate that CESE significantly drives loyalty through the direct path, while PESE enhances loyalty intentions indirectly by supporting the core experience. This highlights the foundational role of core experiences in building brand value, with peripheral experiences offering crucial supplementary support^96,97^. To maximize the impact of core experiences, managers should prioritize technological innovations (e.g., intelligent referee systems, real-time analytics) to improve the fairness and excitement of matches, design event schedules to emphasize competitive tension, and foster emotional resonance through athlete engagement (e.g., autograph sessions, themed fan interactions)^98^. For peripheral experiences, managers can focus on upgrading venue facilities, streamlining service processes (e.g., contactless payment, intelligent navigation), and creating immersive interactive zones (e.g., VR tournament experiences)^99,100^. This “core-driven, environment-enabled” strategy ensures a synergistic optimization of the overall event experience, enhancing both its value and appeal.

Second, SN were found to play a critical mediating role in converting brand experiences into loyalty behaviors, reflecting the importance of social influence mechanisms in collective consumption. Sports events, as highly socialized scenarios, often drive spectator behaviors through group identity and social evaluations. Managers can amplify this effect by building a “digital-physical integrated communication system” to enhance the social impact of brand experiences. Specific measures include pre-event user-generated content (UGC) campaigns (e.g., short video challenges), real-time audience interaction during events (e.g., live polls, quizzes), and post-event online community discussions^101,102^. For female viewers, who are more sensitive to social norms, exclusive events (e.g., women’s themed races) or targeted campaigns on social media platforms (e.g., Instagram, WeChat) can strengthen their sense of community and social identity. This approach not only enhances brand communication but also fosters loyalty behaviors through shared social experiences^83^.

Finally, the significant role of gender differences and individual preferences in influencing brand experience conversion pathways underscores the need for precision marketing strategies. A data-driven audience profiling system, incorporating variables such as gender, interests, and purchasing power, can enable personalized marketing efforts. For instance, creating a “social viewing area” for spectators who value interaction can strengthen social norms via group activities, while a “tactical analysis zone” can cater to professional viewers seeking in-depth insights^103^. For international events, integrating local cultural elements (e.g., traditional cuisine, cultural performances) can enhance global appeal, while for local events, partnerships with community organizations (e.g., fan clubs, volunteer programs) can strengthen local loyalty^104^. This dynamic segmentation approach ensures that diverse audience needs are met effectively, enhancing both communication efficiency and brand impact.

In summary, effective event brand management requires a multi-pronged approach: optimizing the core experience, activating social influence mechanisms, and implementing data-driven precision marketing. By leveraging innovation and audience segmentation, managers can enhance brand value and secure a competitive advantage in the sports event market.

## Future prospects

This study reveals the multi-level mechanism by which SEBE influences SL, but several areas warrant further exploration. First, the sample is limited to Chinese spectators, which may restrict the generalizability of findings. Future research could conduct cross-cultural studies to validate these results and explore how cultural, economic, and demographic differences shape SEBE and SL. Second, the reliance on self-reported, cross-sectional data introduces potential biases (e.g., social desirability bias) and limits the ability to capture the dynamic evolution of brand experiences. Longitudinal studies and advanced tools like AI and Big Data could offer deeper insights into how core and environmental experiences interact and adapt over time. Third, external factors such as marketing strategies, macroeconomic conditions, digital technologies (e.g., VR, metaverse), and sustainability practices were not considered but may significantly impact loyalty pathways. Future research should examine how immersive technologies and green initiatives reshape SEBE and contribute to the digital and sustainable transformation of event branding, advancing theory and providing actionable insights for the evolving sports industry.

## Data Availability

The data that support the findings of this study are available from the corresponding author upon reasonable request.
